# Sensitive and Specific Detection of Ewing Sarcoma Minimal Residual Disease in Ovarian and Testicular Tissues in an In Vitro Model

**DOI:** 10.3390/cancers11111807

**Published:** 2019-11-17

**Authors:** Laure Chaput, Victoria Grèze, Pascale Halle, Nina Radosevic-Robin, Bruno Pereira, Lauren Véronèse, Hervé Lejeune, Philippe Durand, Guillaume Martin, Sandra Sanfilippo, Michel Canis, Justyna Kanold, Andrei Tchirkov, Florence Brugnon

**Affiliations:** 1 Biologie et Médecine de la Reproduction, AMP (Assistance Médicale à la Procréation), CECOS (Centre d’Etude et de la Conservation des Oeufs et du Sperme), CHU (Centre Hospitalier Universitaire) Estaing, CHU Clermont-Ferrand, 63003 Clermont-Ferrand, France; lchaput1@chu-clermontferrand.fr (L.C.); sanfilipposandra@gmail.com (S.S.); 2 Service d’Hématologie et d’Oncologie Pédiatrique, CHU Estaing, CHU Clermont-Ferrand, 63000 Clermont-Ferrand, France; vgreze@chu-clermontferrand.fr (V.G.); jkanold@chu-clermontferrand.fr (J.K.); 3 INSERM-CIC 1405 (Institut national de la santé et de la recherche médicale-Centre d’investigation clinique), Unité CRECHE (Centre de recherche clinique chez l’enfant), 63001 Clermont-Ferrand, France; 4 Centre de Biothérapie d’Auvergne, CHU Estaing, CHU Clermont-Ferrand, 63000 Clermont-Ferrand, France; phalle@chu-clermontferrand.fr; 5 Laboratoire de Biologie Médicale (LBM) OncoGenAuvergne, Unité Pathologie, Centre Jean Perrin (CLCC de la région Auvergne), 63011 Clermont-Ferrand, France; nina.robin@cjp.fr; 6 Imagerie Moléculaire et Stratégies Théranostiques Faculté de Médecine, Unité INSERM 1240, Université Clermont Auvergne, 63001 Clermont Ferrand, France; lveronese@chu-clermontferrand.fr (L.V.); atchirkov@chu-clermontferrand.fr (A.T.); 7 Délégation à la Recherche Clinique et l’Innovation, Unité de Biostatistiques, CHU Clermont-Ferrand, 63000 Clermont-Ferrand, France; bpereira@chu-clermontferrand.fr; 8 Service de Cytogénétique Médicale, CHU Clermont-Ferrand, 63000 Clermont-Ferrand, France; 9 Service de Médecine de la Reproduction, Hôpital Femme Mère Enfant, Hospices Civils de Lyon, 69003 Lyon, France; herve.lejeune@chu-lyon.fr; 10 Unité INSERM U 1208 Laboratoire de Biologie de la Reproduction, Université Claude Bernard Lyon 1, 69100 Villeurbanne, France; 11 Kallistem, Ecole Normale Supérieure de Lyon, 69007 Lyon, France; philippe.durand@kallistem.com (P.D.); guillaume.martin@kallistem.com (G.M.); 12 Service de Gynécologie, CHU Estaing, CHU Clermont-Ferrand, 63000 Clermont-Ferrand, France; mcanis@chu-clermontferrand.fr

**Keywords:** fertility preservation, Ewing sarcoma, ovarian tissue, testicular tissue, minimal residual disease detection, RT-qPCR

## Abstract

Ewing sarcoma (EWS) is a common pediatric solid tumor with high metastatic potential. Due to toxic effects of treatments on reproductive functions, the cryopreservation of ovarian tissue (OT) or testicular tissue (TT) is recommended to preserve fertility. However, the risk of reintroducing residual metastatic tumor cells should be evaluated before fertility restoration. Our goal was to validate a sensitive and specific approach for EWS minimal residual disease (MRD) detection in frozen germinal tissues. Thawed OT (*n* = 12) and TT (*n* = 14) were contaminated with tumor RD-ES cells (10, 100, and 1000 cells) and EWS-FLI1 tumor-specific transcript was quantified with RT-qPCR. All contaminated samples were found to be positive, with a strong correlation between RD-ES cell numbers and EWS-FLI1 levels in OT (*r* = 0.93) and TT (*r* = 0.96) (*p* < 0.001). No transcript was detected in uncontaminated control samples. The invasive potential of Ewing cells was evaluated using co-culture techniques. After co-culturing, tumor cells were detected in OT/TT with histology, FISH, and RT-qPCR. In addition, four OT and four TT samples from children with metastatic EWS were tested, and no MRD was found using RT-qPCR and histology. We demonstrated the high sensitivity and specificity of RT-qPCR to detect EWS MRD in OT/TT samples. Clinical trial: NCT 02400970.

## 1. Introduction

Ewing sarcoma (EWS) is one of the most common solid tumors in children and adolescents but also in young adults [1], and is characterized by a high metastatic dissemination potential [2,3,4]. EWS is the second most common bone cancer in children [5]. The study of Schleiermarcher et al. [6] reported that 27% of patients had evidence of metastasis at diagnosis. EWS metastases spread hematogenously to lungs, bones, bone marrow, and in some cases ovaries [7]. The treatment of EWS most often requires a combination of multi-agent chemotherapy, sometimes followed by hematopoietic stem cell transplantation, which are known to be sterilizing treatments. Patients treated with pelvic radiotherapy have a high risk of premature ovarian insufficiency. Moreover, the risks of premature ovarian insufficiency and infertility are directly proportional to the received cumulative dose of alkylating agents and pelvic radiotherapy [8]. Fertility preservation by cryoconservation of germinal tissues and gametes of EWS patients is therefore recommended. Ideally, fertility preservation should take place before the first cycle of chemotherapy by cryoconservation of germinal tissues [9]. For prepubertal children, the option chosen for fertility preservation is the freezing of ovarian and testicular tissues (OT and TT). Today more than 86 births have been reported after grafts of frozen OT [10]. For males, testicular stem cell transplantation into seminiferous tubules to enable spermatogenesis has recently been reported in a macaque model [11] or in vitro maturation [12]. Restoration for fertility is thus possible for females and foreseeable for males. However, there may be malignant cells in germinal tissue in metastatic EWS, with a consequent risk of transplanting tumor cells with cryopreserved tissues. It is therefore essential to be able to detect and assess minimal residual disease (MRD) in germinal tissue from such patients.

In 85–90% of cases, EWS tumors are characterized by a specific chromosomal translocation, t(11;22)(q24;q12), resulting in the fusion of the EWSR1 (22q12) gene and a member of the ETS family or transcription factor FLI1 (11q24) gene. This fusion transcript can be detected with a high sensitivity and specificity by RT-qPCR (reverse transcriptase-polymerase chain reaction) and used for MRD evaluation [2,6,13]. EWS MRD detection by RT-PCR has been performed in OT. Schifflers et al. [14] studied MRD in OT from patients with non-metastatic EWS. Abir et al. described EWS MRD detection of cryopreserved ovarian samples by snap freezing [15]. The immunochemistry detection for CD99 and histology were negative and the RT-qPCR (EWS-FLI1 transcript) was positive in one patient out of five tested. Another study evaluated the presence of malignant cells in one piece of OT cryopreserved by slow freezing [16]. Histology and quantitative RT-PCR were performed before and after xenotransplantation to immunodeficient mice and showed negative results. Of note, no MRD evaluation in TT has been reported.

To assess the accuracy of EWS MRD detection, we quantified EWS-FLI1 transcript in human OT and TT cryopreserved by slow or snap freezing and contaminated in vitro with increasing numbers of EWS tumor cells using the RD-ES cell line. We evaluated the dissemination potential of RD-ES cells within germinal tissue to validate our in vitro model. MRD detection was then performed in frozen OT and TT samples from EWS patients. To our knowledge, this is the first MRD study comparing two freezing protocols in OT and TT samples and investigating EWS contamination in testicles.

## 2. Results

### 2.1. Experimental Model of EWS Contamination of Germinal Tissues

Thawed OT (*n* = 12 contamination series) enclosing ovarian cysts from women with benign cysts, and thawed TT (*n* = 14 contamination series) from patients with azoospermia were contaminated with 0 (negative control), 10, 100, and 1000 human EWS cells (RD-ES cell line). Quantification of EWS-FL1 transcript of type II expression was performed in thawed OT and TT. After contamination, RNA extraction and RT-qPCR were performed. OT and TT frozen by either slow or snap freezing methods were used to determine whether the freezing method could interfere with RT-qPCR analysis. To ensure that RD-ES cell lines had a similar dissemination potential compared to in vivo conditions, we co-cultured OT and TT with RD-ES cells. After 7 and 14 days of co-culture we performed RT-qPCR, histological analysis and FISH analysis in germinal tissues to look for dissemination of RD-ES cells. We analyzed TT by immunochemistry with ERG staining.

### 2.2. Yield of RNA Extraction from Germinal Tissues Frozen Using Slow or Snap Freezing

The median weight of OT fragments was 37.4 mg [15.2–62.0 mg]. OT was frozen by slow freezing (*n* = 6) or snap freezing (*n* = 6). The median weight of TT fragments was 28.8 mg [16.6–48.0]. TT was frozen by slow freezing (*n* = 7) or snap freezing (*n* = 7). After freezing by slow and snap freezing, the RNA yields extracted from OT (30.4 µg vs. 19.8 µg) and TT (19.8 µg vs. 22.8 µg) were not significantly different according to the freezing method (*p* > 0.05).

### 2.3. Detection of EWS-FLI1 Transcript in Frozen OT and TT Samples Contaminated with RD-ES Cells

**Figure 1:**
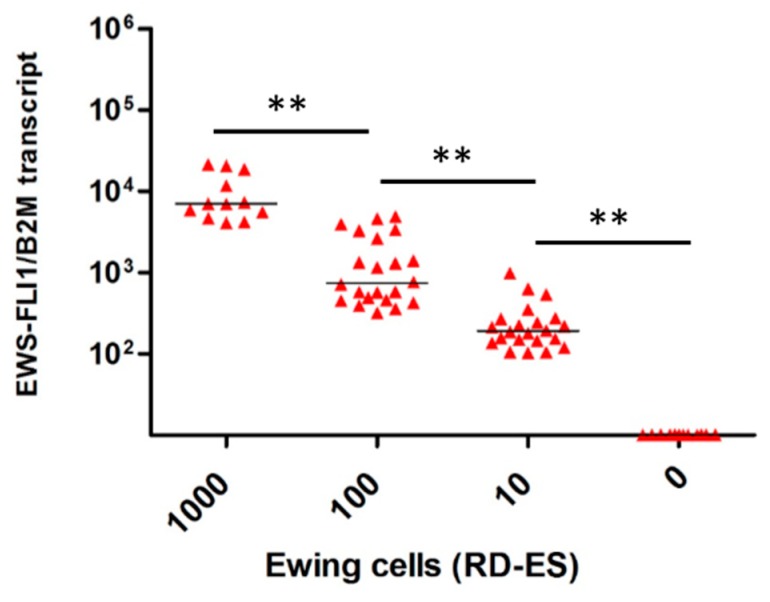
Ewing sarcoma (EWS)-FLI1 transcripts detection in ovarian tissue (*n* = 12). Relative quantification of *EWS-FLI1* transcripts (B2M reference gene) for the contamination with 0, 10, 100 and 1000 cells. Each symbol represents one ovarian fragment (the average of the duplicates for 1000 cells or triplicates for 10 and 100 cells). The symbol ** means there was a significant difference and *p* < 0.001.

**Figure 2:**
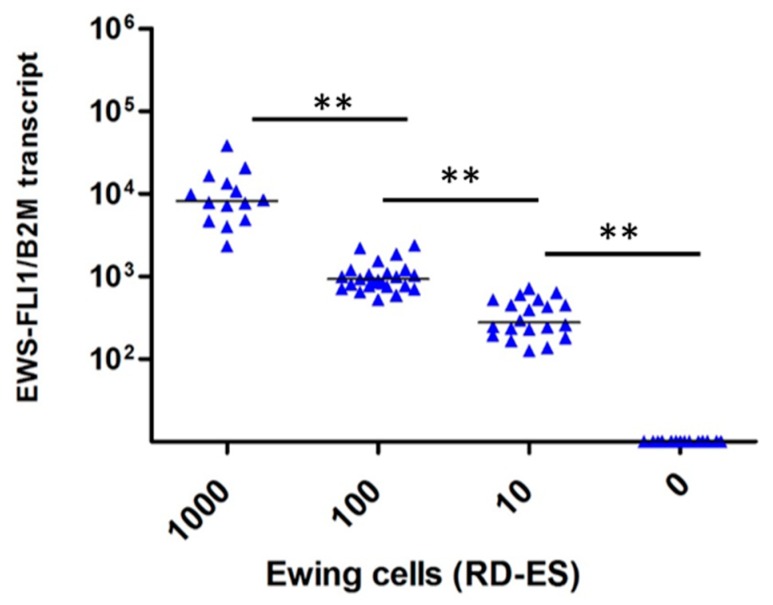
EWS-FLI1 transcripts detection in testicular tissue (*n* = 14). Relative quantification of *EWS-FLI1* transcripts (B2M reference gene) for the contamination with 0, 10, 100, and 1000 cells. Each symbol represents one testicular fragment (the average of the duplicates for 1000 cells or triplicates for 10 and 100 cells). The symbol ** means there was a significant difference and *p* < 0.001.

**Figure 3:**
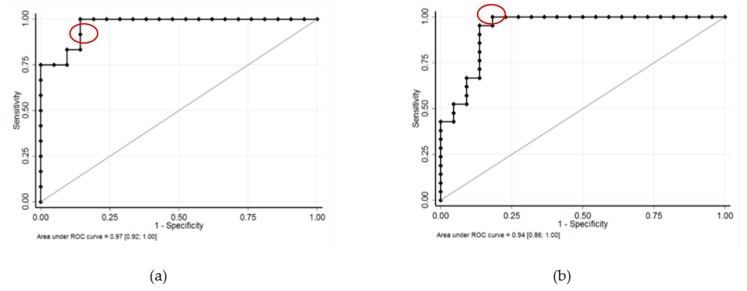
Sensitivity (SE) and specificity (SP) of detection to distinguish 10 and 100 Ewing cells, and 100 and 1000 Ewing cells in ovarian tissue: (**a**) The AUC (area under the curve, ROC curve) was 0.94 CI 95% [0.86–1.00] to distinguish 10 and 100 Ewing cells. The optimal decision threshold, determined using Liu and Youden indexes, to distinguish between 10 and 100 EWS cells was 354 EWS-FLI1 transcripts with a sensitivity (SE) of 95% and a specificity (SP) of 86% (in red). For maximal SE (100%) and SP (100%), the cut-offs were 319 and 1150 EWS-FLI1 transcripts, respectively. (**b**) The area under the curve (AUC) was 0.97 CI 95% [0.92–1.00] between 100 and 1000 Ewing cells. To distinguish between 100 and 1000 EWS cells, the optimal decision threshold determined using Liu and Youden indexes was 3998 EWS-FLI1 transcripts with a SE of 100% and SP of 86% (in red). For a maximal SP (100%), the cut-off was 5528 EWS-FLI1 transcripts.

**Figure 4:**
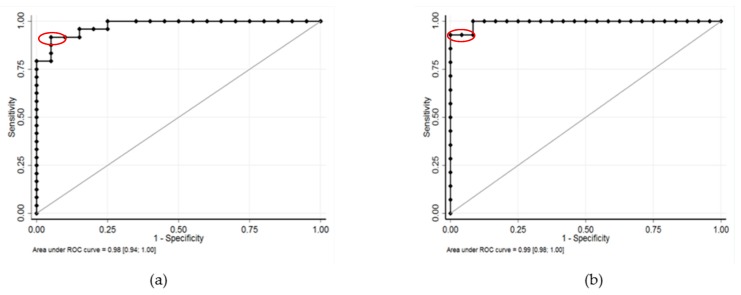
Sensitivity and specificity of detection to distinguish 10 and 100 EWS cells, and 100 and 1000 EWS cells in testicular tissue: (**a**) The AUC was 0.98 CI 95% [0.94–1.00] to characterize 10 and 100 EWS cells. The thresholds to distinguish between 10 and 100 EWS cells were 642 EWS-FLI1 transcripts (Liu and Youden indexes, SE = 92% and SP = 95%) (in red), 521 EWS-FLI1 transcripts for SE = 100% and 749 EWS-FLI1 transcripts for SP = 100%. (**b**) The AUC was 0.99 CI 95% [0.98–1.00] between 100 and 1000 EWS cells. The cut-offs to distinguish between 100 and 1000 EWS were 3172 EWS-FLI1 transcripts (Liu and Youden indexes, SE = 93% and SP = 100%) (in red) and 2170 EWS-FLI1 transcripts for SE = 100%.

No expression of EWS-FLI1 transcript was observed in uncontaminated OT (*n* = 12) and TT (*n* = 14) frozen by slow or snap freezing. EWS-FLI1 transcript was detected in all contaminated OT (Figure 1) and TT (Figure 2). A close correlation between the number of RD-ES cells (10, 100, and 1000 cells) and EWS-FLI1 transcript was observed in OT (*r* = 0.93, *p* < 0.001) and in TT (*r* = 0.96, *p* < 0.001). We studied the sensitivity and specificity of the Ewing MRD detection by RT-qPCR. For OT, the AUCs (area under the curve, ROC curve) were 0.94 to distinguish 10 and 100 EWS cells CI 95% [0.86–1.00] (Figure 3a) and 0.97 CI 95% [0.92–1.00] between 100 and 1000 EWS cells (Figure 3b). For TT, the AUCs were respectively 0.98 to characterize 10 and 100 EWS cells CI 95% [0.94–1.00] (Figure 4a) and 0.99 CI 95% [0.98–1.00] between 100 and 1000 EWS cells (Figure 4b).

### 2.4. Measurements of Dissemination Potential of RD-ES Cell Lines After Co-Culture with Ovarian and Testicular Samples

**Figure 5:**
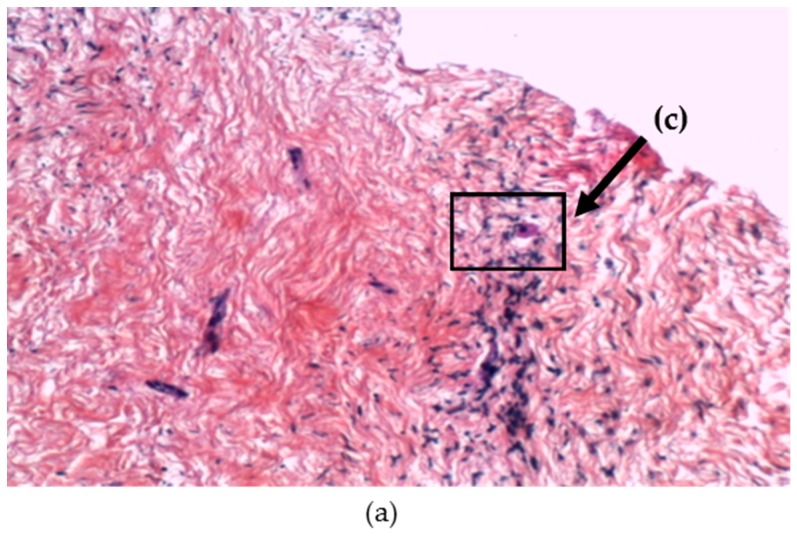

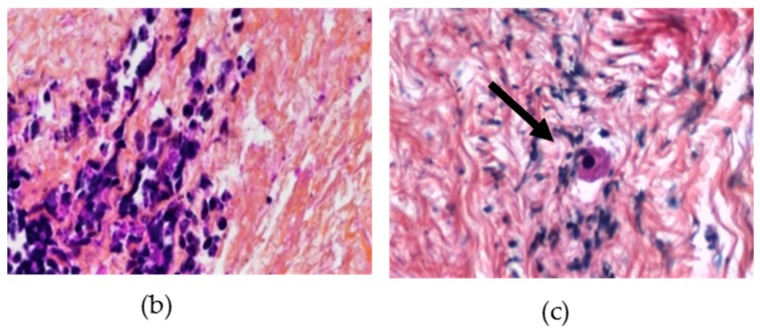
Illustrations of histology: ovarian tissue (OT) after co-culture with RD-ES cells (at day 7); (**a**) RD-ES cells (arrow) localized in OT after co-culture stained with hematoxylin and eosin (×10); (**b**) Area with RD-ES cells disseminated in OT (×20); (**c**) Area with ovarian follicle (×20).

**Figure 6:**
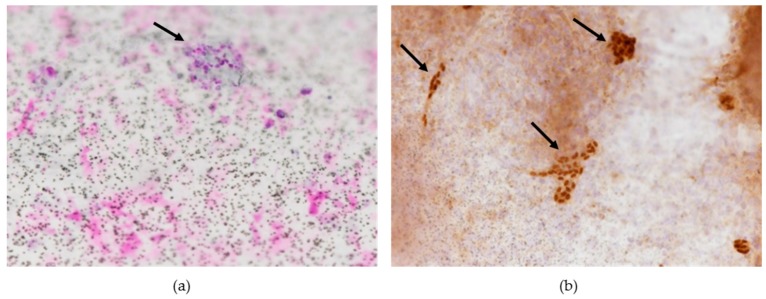
Illustrations of histology and immunohistochemistry: RD-ES cells (black arrow) localized in testicular tissue (TT) after co-culture (at day 14) on insert; (**a**) TT in insert stained with hematoxylin and eosin (×20); (**b**) ERG-positive staining of RD-ES cells (×20).

**Figure 7:**
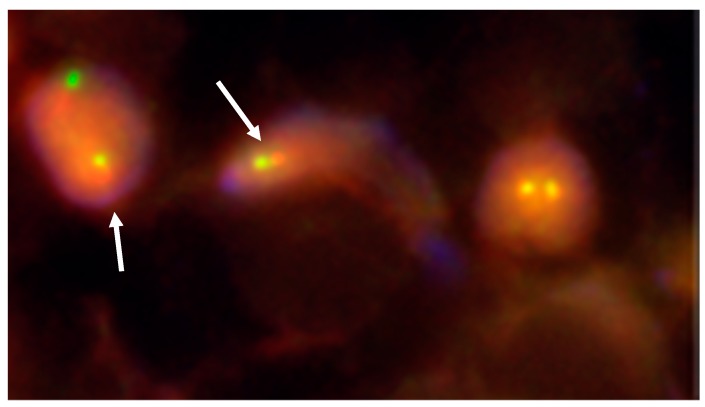
Fluorescence in situ hybridization (FISH) analysis of ovarian tissue using EWSR1 (22q12) dual color break apart rearrangement probe (Vysis) showing RD-ES cells invasion (white arrows) after co-culture (at day 14) (×80). RD-ES cells displayed one fusion (yellow signal), and the simultaneous split pattern of one orange and one green signal (arrows), indicative of a rearrangement of one copy of the EWSR1 gene. The fusion gene is detected by a yellow signal, corresponding to co-localization of the red and green probes.

EWS-FLI1 transcripts were detected at days 7 and 14 of co-culture in OT and TT. At days 7 and 14, we observed RD-ES cells disseminated in the ovarian tissue (Figure 5) and in the testicular tissue with typically small, round, blue cell morphology after hematoxylin and eosin (H & E) staining (Figure 6a). Using FISH, we detected Ewing cells by the presence of a split signal pattern (EWSR1 rearrangement) in OT (Figure 7). FISH could not be performed on TT co-culture due to technical limits (specific design of culture chambers). After immunohistochemistry, the ERG intra-nuclear staining of Ewing cells was positive after days 7 and 14 of TT co-culture with RD-ES cells (Figure 6b). These results demonstrated the dissemination potential of EWS cells in our in vitro model of tumor contamination of germinal tissue.

### 2.5. MRD Analysis in Ovarian and Testicular Tissues of Patients with EWS

We analyzed cryopreserved OT (*n* = 5) and TT (*n* = 3) from EWS patients using RT-qPCR (Table 1). Most of the patients had received chemotherapy before cryopreservation. Only one boy was not treated prior to TT preservation. The analysis did not detect the presence of EWS-FLI1 transcript. B2M expression was detected at similar levels in OT and TT in the in vitro model described previously, indicating good quality of RNA samples. No EWS cells were detected by standard histological analysis and FISH in OT.

## 3. Discussion

Given the risk of infertility after cancer treatment for EWS [17], fertility preservation by OT and TT cryoconservation is recommended for prepubertal patients. EWS is an aggressive and highly metastatic cancer driven by the expression of the EWS-FLI1 fusion oncogene resulting from a chromosomal translocation [5]. Development of sensitive and accurate methods for detecting metastatic EWS cells in germinal tissue is essential to assess the risk of reintroducing cancer when thawed tissue is used to restore fertility and/or induce puberty in patients [18,19,20]. In the present study, an in vitro model of EWS dissemination in human frozen OT and TT was established and its sensitivity and specificity for the detection of EWS-FLI1 transcript evaluated.

The detection of EWS-FLI1 fusion transcript expression by RT-qPCR is a rapid, specific, and sensitive (1/10^6^ cells) diagnostic test that has mainly been applied for MRD detection in peripheral blood and bone marrow [21]. The RT-PCR assay for detecting sarcoma translocations in tumor tissue is also of clinical utility in differentiating small round blue cell tumors [22]. EWS-FLI1 translocation is detectable with both RT-PCR and FISH in formalin-fixed paraffin-embedded tissue, but FISH seems more reliable than RT-PCR for the diagnosis of EWS in this type of sample [23]. Immunohistochemical markers can assist in differential diagnosis, but the current panels have limited specificity. For this reason, the use of FISH is preferable [22]. FISH and RT-PCR are complementary, and the use of both techniques is needed to clarify the most diagnostically challenging cases [23].

In our in vitro contamination model, we demonstrate the high sensitivity and specificity of the EWS-FLI1 transcript detection in frozen-thawed OT and TT. The histology of ovarian and testicular tissues used in our in vitro model are similar to that of these patients. The ovarian tissue used in this study surrounded benign cysts and contained mainly primordial and primary follicles. The different types of cells (mainly spermatogonia, spermatocytes, and some spermatids/spermatozoa) contained in testicular tissues used in our model came from azoospermia patients and therefore were close to the histology of the EWS patients. Previous studies reported MRD detection in OT but did not evaluate the sensitivity and specificity of the detection. To our knowledge, our study is the first validation of EWS MRD detection in TT. It demonstrates that EWS assessment in OT and TT by RT-qPCR is highly specific, sensitive, and accurate. Although our in vitro model lets us check precisely the numbers of RD-ES cells used for the contamination, it does not enable us to evaluate the accuracy of MRD detection of tumour cells processed under the same conditions as ovarian and testicular tissue, i.e., frozen and thawed. We also show that RD-ES cells used in our in vitro model had high dissemination potential in co-cultures with germinal cells. EWS infiltration was detected by RT-qPCR in OT and TT, by FISH in OT, and Ewing round blue cells were highlighted by histology and immunochemistry (ERG staining) in TT. FISH could not be performed on TT co-culture owing to technical limits set by the specific design of culture chambers. This co-culture of human seminiferous tubules and RD-ES cells was run with previously defined and validated conditions [12].

Sufficient RNA amounts were extracted from small pieces of ovarian and testicular samples independently of the freezing method applied to germinal tissues. To our knowledge, this is the first study to compare EWS MRD detection in germinal tissues frozen by two protocols. Our results show that the freezing method had no impact on RNA yields. RT-qPCR can therefore be performed on OT and TT samples that have been already cryopreserved using slow freezing and on samples that will be frozen by snap freezing at the time of surgical retrieval for subsequent analysis. The use of small OT and TT samples for MRD analysis ensures that sufficient amounts of tissue remain for fertility preservation.

The incidence of ovarian or testicular metastases in EWS is not well known. Circulating tumor cells can be detected in 20–45% of EWS patients [6]. This suggests that small numbers of malignant cells may be present in ovary [16] and testis, as well as in other known metastatic sites for EWS such as lung, bone, and bone marrow [6]. At diagnosis, 20–30% of patients show evidence of metastasis that involves mainly the lung, bone, and bone marrow [6]. Cases of metastatic ovarian involvement in EWS patients have been described in the literature [3,24]. Microscopic ovarian infiltration could be present in non-metastatic EWS [14]. No metastasis in TT has been described [25]. In our study, MRD was not detected in germinal tissues of patients with metastatic or localized EWS. However, the germinal tissues we analyzed were obtained after chemotherapy.

Previously, EWS MRD was studied in ovarian samples with different approaches. In the study of Abir et al. one ovarian sample cryopreserved by snap freezing was evaluated from each EWS patient [15] and no malignant cell was detected by histology, but RT-PCR showed a positive result for the transcript *EWS–FLI1* in one case. Greve et al. investigated the presence of malignant cells in one piece of OT intended for transplantation [16]. All the samples underwent histology and RT-qPCR and none of the pieces revealed Ewing cells or expression of EWS–FLI1 transcripts. These results suggest that MRD needs to be evaluated by different techniques before ovarian transplantation. In our study, we performed RT-qPCR, FISH, and histology to combine more detection methods. In the case of MRD positivity, it may be feasible to eliminate tumor cells by grafting isolated ovarian follicles or by performing in vitro maturation of oocytes to ensure safer transplantation. Such methods have already produced live offspring in mice [26] and mature oocytes in primates [27]. In such cases, it may be feasible to graft isolated ovarian follicles or to perform in vitro maturation of oocytes to ensure safer transplantation. This would eliminate the risk of transplanting malignant cells hidden in ovarian stromal tissue. Such methods have already produced live offspring in mice [26] and mature oocytes in primates [27].

## 4. Materials and Methods

### 4.1. Patients and Samples

Written informed consent was obtained from all patients for sample inclusion in the Germetheque biobank with the approval of the Committee for Personal Protection (AC 2009-886). The study was declared on the clinicaltrial.gov website (No. NCT 02400970).

OT was collected from ten patients (mean age 31.4 ± 7.6 years, range 23–42 years) during endoscopic surgery for benign ovarian cysts at the University Hospital of Clermont-Ferrand, France between January 2017 and July 2017. For each patient, a piece of ovarian cortex overlying the cyst was excised using scissors and without electrocoagulation.

TT was obtained from eight patients (mean age 36.5 ± 4.7 years, range 30–46 years) with non-obstructive azoospermia between January 2017 and July 2017 at the University Hospital of Clermont-Ferrand, France. These men underwent testicular sperm extraction for intra-cytoplasmic sperm injection (ICSI).

In addition, OT and TT obtained from respectively five girls (mean age 15.8 ± 1.8 years, range 13–16 years) and three boys (mean age 11.6 ± 4.0 years, range 7–14 years) with EWS were analyzed to measure the expression of potential MRD. Each patient had positive detection of EWS-FLI1 type II in the primitive tumor.

### 4.2. Freezing and Thawing of Germinal Tissue Samples

The samples were first cut into six thin fragments of equal size (1–2 mm^3^) after weighing. OT and TT were frozen by slow freezing or snap freezing to compare the two methods. The slow freezing method was performed on OT and TT as previously described respectively by Sanfilippo et al. [28] and Rives et al. [29]. For the snap freezing method, OT and TT samples were placed in cryogenic vials (Cryo Bio System, IMV Technologies Group, L’Aigle, France) and immediately frozen by immersion in liquid nitrogen. The cryovials were immersed in liquid nitrogen for storage.

The same thawing procedure was used for OT frozen by slow or snap freezing. Cryovials were immersed in a water bath (37 °C, 2 min) and each sample was washed twice in medium A for 5 min at 37 °C. The same thawing procedure was used for TT frozen by slow or snap freezing. Cryogenic vials were thawed in a water bath at 37 °C for 3 min and then incubated in two baths of Leibowitz L15 medium (Eurobio, Courtaboeuf, France) for 5 min at 37 °C.

### 4.3. Culture of RD-ES Subclones and Contamination

We used well-established RD-ES human Ewing sarcoma cell lines (RD-ES, ATCC LGC Standards, Molsheim, France). These cells were maintained in RPMI (Roswell Park Memorial Institute Medium, Fisher Scientific, Illkirch, France) supplemented with 15% fetal calf serum (Fisher Scientific), 1% l-glutamine (Sigma Aldrich, St. Quentin Fallavier, France), and 1% antibiotics (penicillin-streptomycin, Sigma Aldrich) at 37 °C. Standard cell counts and blue trypan staining for viability were performed prior to each tissue contamination. The viability of RD-ES cells exceeded 80%.

After thawing, fragments of OT (*n* = 12 contamination series) and TT (*n* = 14 contamination series) with respective median weights 37.4 mg [15.2–62.0] and 30.0 mg [16.6–48.0] were contaminated with 10, 100, and 1000 RD-ES cells. One fragment was not contaminated (negative control). Contaminations with 10 and 100 RD-ES cells were assessed in duplicate. The positive control was validated for each contamination series by RT-qPCR analysis of 10^6^ RD-ES cells.

### 4.4. Detection of EWS-FLI1 of Type II mRNA Expression by RT-qPCR

Total RNA was extracted using the TRIzol reagent (Invitrogen, Cergy-Pontoise, France) following the manufacturer’s instructions. RNA concentration was evaluated using a UV-visible spectrophotometer (Nanodrop 2000C, Thermo Scientific, Illkirch, France). RNA was then treated with DNase I (Roche Diagnostics, Meylan, France) and reverse transcribed using the Superscript II enzyme (Invitrogen, Cergy-Pontoise, France). Complementary DNA was purified using a Qiagen^®^ silica column (Qiagen, Courtaboeuf, France) to eliminate possible PCR inhibitors.

PCR quantification was performed on the LightCycler 480 Real Time PCR system, (Roche Diagnostics, Meylan, France). EWS-FLI1 type II transcript detection was performed using previously described forward (5′-CCAAGTCAATATAGCCAACAG-3′), reverse (5′- GGCCAGAATTCATGTTATTGC-3′) primers and probe (FAM5′-ACGGGCAGCAGAACCCTTCTTAT-3′TAMRA) [22]. The reference β-2-microglobulin (β2M) gene transcript was detected using the following forward (5′- GAGTATGCCTGCCGTGTG-3′) and reverse (5′-AATCCAAATGC-GCATCT-3′) primers and probe (FAM5′-CCTCCATGATGCTGCTTACATGTCTC -3′-TAMRA). A two-step amplification was performed using an initial denaturation step (95 °C for 5 min) followed by 50 cycles of 95 °C for 15 s and 60 °C for 1 min. All PCR reactions were performed in triplicate. Copy numbers of EWS-FLI1 transcripts were reported per 10^6^ copies of β2M transcripts.

### 4.5. Detection of Potential Dissemination of RD-ES Cells in OT and TT After co-Cultures

#### 4.5.1. OT Co-Culture

After thawing, small pieces of OT (1 mm^3^) were individually transferred to low attachment 96-well plates (Corning, Escolab, Belgium). These OT fragments were subsequently cultured in pre-equilibrated α-Minimum Essential Medium containing GlutaMAX (Invitrogen, Belgium) and supplemented with HSA (10%, Vitrolife, Göteborg, Sweden), ascorbic acid (100 μg/mL), insulin (5 ng/mL), transferrin-selenium (5 μg/mL–5 ng/mL), and recombinant FSH (25 mIU/mL, GONAL-f, Merck, Lyon, France). Culture lasted 14 days (37 °C, humidified incubator, 5% CO_2_). 100,000 RD-ES cells were then added to each culture well.

After co-cultures (on days 7 and 14), we performed RT-qPCR (as previously described), histologic analysis and FISH analysis.

##### Histologic Analysis

The co-cultured OT was fixed in 4% formalin and embedded in paraffin (FFPE). The 3 µm-thick sections were cut out of the FFPE blocks, stained with hematoxylin and eosin (H & E), and examined under a bright-field microscope.

##### Fluorescence in Situ Hybridization (FISH) after Co-Cultures of Ovarian Tissue and RD-ES cells

FISH was performed to detect EWSR gene rearrangement using Vysis LSI EWSR1 Break Apart FISH probe kit (Abbott Molecular, Abbott Park, IL, USA) in accordance with the manufacturer’s recommended procedures as described previously [30]. With pepsin for 25 min at 37 °C, slides were deparaffinized and immersed in ethanol baths. After air_drying, hybridization was performed. After counterstaining with 4′-6-diamidino-2-phenylindole dihydrochloride (DAPI). Images were acquired with an Axioplan2 imaging fluorescence microscope (ZEISS, Göttingen, Germany) equipped with a CCD camera and appropriate filters. Images were subsequently analyzed with the digital-imaging software Isis v3.8.8 (MetaSystems, Altlussheim, Germany).

The orange and green probes used for this analysis labelled the 5’ and the 3’ site of the EWSR1 gene, respectively. Therefore, each allele of the EWSR1 gene will exhibit two contiguous orange and green signals in cells with intact 22q12 regions. On the contrary, in the presence of translocations related to the 22q12 region, the two signals will be split. We considered an allele positive for EWSR1 rearrangement when the gap between the green and orange signals exceeded the diameter of either one.

#### 4.5.2. TT Co-Culture

TT was obtained from bilateral orchiectomy performed for gender reassignment of one transgender subject aged 24 years (Prof. H. Lejeune) at the University Hospital of Lyon, France. The protocol was approved by the local research ethics committee (Comité d’éthique des Hospices Civils de Lyon). The co-culture was carried out after adaptation in humans of published conditions in rats [12]. Isolated seminiferous tubules (20–50 mm^3^) after enzymatic digestion and mechanical dissociation were inoculated into a bicameral chamber with Bio-Alter^®^ technology. Thirty-thousand RD-ES cells were then added to each culture well.

After co-cultures (on days 7 and 14), we performed RT-qPCR (as previously described), histologic analysis and immunochemistry (ERG immunoreactivity) in TT (anti-ERG monoclonal mouse antibody, clone 9FY, Zytomed Systems GmbH, Berlin, Germany).

##### Immunohistochemistry (IHC)

Inserts containing co-culture testicular tissue and the RD-ES cells were fixed in buffered neutral 10% formalin and glued on glass slides for IHC (Dako FLEX IHC Microscope Slides, Agilent Technologies France, Les Ullis, France). After antigen unmasking for 45 min at 98 °C in CC1 buffer (Cell Conditioning 1, Ventana/Roche), incubation with anti-ERG monoclonal antibody (anti-ERG monoclonal mouse antibody, clone 9FY, Zytomed) was performed overnight at 4 °C in a humid chamber. The antigen-antibody reaction was revealed with Dako REAL kit DAB, according to the manufacturer’s instructions. The presence of granular brown deposits in the nuclei of Ewing sarcoma cells was considered positive staining. Interpretation of the IHC staining was performed by two researchers independently (Laure Chaput, Nina Robin). If they diverged, consensus was reached by conferral with a multi-head microscope (Axiophot 2, Zeiss, Oberkochen, Germany).

### 4.6. Statistical Analysis

Statistical analyses were performed using Stata software (version 13, StataCorp, College Station, TX, USA). The tests were two-sided with a type I error at 5%. Continuous parameters were described, according to statistical distribution, as mean ± standard deviation or median (interquartile range). The assumption of normality was studied using Shapiro-Wilk’s test. The study of relationships between continuous parameters was analyzed estimating Pearson or Spearman correlation coefficients (noted *r*). Due to several measurements for the same patient, usual statistical tests were inappropriate because the hypothesis of data independence was not met. Also, random-effects models for correlated data were carried out to take into account between- and within-subject variability. The assumption of residuals normality was studied. When appropriate, a logarithmic transformation was used to achieve the normality of the dependent variable, and to guarantee the correct use of aforementioned analyses. A Šidák type I error correction was applied to allow for multiple comparisons. Finally, a ROC (receiver operating characteristic) curve analysis was carried out to evaluate the sensitivity and specificity of EWS-FLI1 transcript detection (RT-qPCR) in OT and TT samples. The confidence intervals at 95% were presented for area under the curve (AUC), sensitivity and specificity.

## 5. Conclusions

In conclusion, we showed with our in vitro model that EWS-FLI1 mRNA detection by qPCR provides an accurate assessment of EWS MRD in ovarian and testicular tissues cryopreserved by slow or snap freezing. Since EWS is highly metastatic, MRD detection of EWS always needs to be confirmed by additional methods (histology, FISH and/or immunostaining) to ensure there is no risk of reintroducing cancer cells. These results now require confirmation by a further study of ovarian and testicular tissues from a larger cohort of prepubertal patients with EWS.

## Figures and Tables

**Table 1:** EWS patient characteristics (OT: Ovarian Tissue; TT: Testicular Tissue).

EWS Patients	Age at Cryopreservation (Years)	Chemotherapy	Metastasis	Outcome
OT A	16	Before preservation	Lung	Dead
OT B	14	Before preservation	Lung	Alive
OT C	14	Before preservation	No	Alive
OT D	15	Before preservation	Lung, bone and medullary	Dead
OT E	13	Before preservation	Lung	Alive
TT A	7	Before preservation	Mediastinum	Alive
TT B	14	Before preservation	None	Alive
TT C	14	After preservation	None	Alive
